# Quick Monitoring of Tomato and Onion Samples During Routine Regulatory Analysis of Pesticide Residues

**DOI:** 10.1007/s00244-025-01133-w

**Published:** 2025-06-14

**Authors:** José Manuel Veiga-del-Baño, José Oliva, Miguel Ángel Cámara, Pedro Andreo-Martínez, Miguel Motas

**Affiliations:** 1 Department of Agricultural Chemistry, Faculty of Chemistry, Regional Campus of International Excellence “Campus Mare Nostrum”, University of Murcia, Campus of Espinardo, 30100 Murcia, Spain; 2 Department of Toxicology, Faculty of Veterinary, Regional Campus of International Excellence “Campus Mare Nostrum”, University of Murcia, Campus of Espinardo, 30100 Murcia, Spain

## Abstract

In this study, a rapid visualization method was developed to simultaneously evaluate the on-going performance of routine analysis and ensure that the concentration of multiple pesticides in food samples with high water content complies with Maximum Residue Limits (MRLs). In order to accomplish this, we used a bubble chart known as Fast Risk Estimation and Analysis. In this chart, each pesticide is represented by a bubble. By looking at its color, position on the graph, and the size of the bubble, you can quickly determine whether it meets the requirements for relative standard deviation (RSD), recovery, Index of Quality for Residues, and matrix effect. Onion and pepper were chosen as commodity group with high water content. A single matrix-matched calibration using pepper was performed to analyze all these products. The risk visualization allows simultaneous checking of on-going validation and quality control parameters as recovery between 60 and 140% of the spike samples analyzed at the same time, such as the historical RSD (value and alarm if is upper at 20%). At the same time, the bubble chart monitoring other parameters such as the exceeding of the MRL in the analyzed samples, the complex definition of the residue in different pesticides or inconsistencies such as limits of quantification higher than the MRL could be quickly identified.

**Supplementary Information:**

The online version contains supplementary material available at 10.1007/s00244-025-01133-w.

Pesticides are extensively utilized to safeguard livestock and crops in the agricultural sector. For example, in 2021, approximately 333,000 tons of pesticides were used, along with the introduction of over 400 new pesticides to the market (Eurostat 2023).

When these pesticides are used on crops, it is important to ensure that only a minimal amount of them ends up in the food supply. To address this, Maximum Residue Limits (MRLs) have been established by regulations worldwide to prevent health issues linked to the overuse of pesticides (José Manuel Veiga-del-Baño et al. 2023). In the European Union (EU), for instance, the European Commission’s (EC) consolidated version of Regulation 396/2005 sets MRLs for a range of foodstuffs (EC 2005; Kuchheuser and Birringer 2022).

The main goal of pesticide analysis is to develop effective methods for assessing food safety that comply with international quality standards (SANTE 2021). To determine as many pesticides as possible in the most cost-effective way with the least effort, Multi-Residue Methods are needed. For this reason, pesticides are determined by gas chromatography (GC) and liquid chromatography (LC) coupled with mass spectrometry (MS), and the most widely used pesticide residue extraction method in routine laboratories today is the method with the acronym “Quick, Easy, Cheap, Effective, Rugged, and Safe” (QuEChERS). The QuEChERS method involves two steps, the first being an initial extraction with different salt formulations to drive the separation of the organic extraction solvent and water. The second, where an aliquot of the organic phase goes through a clean-up process in dispersive solid phase extraction (d-SPE) using different sorbents to remove the matrix component prior to injection by chromatographic analysis (Anastassiades et al. 2002; José M. Veiga-del-Baño et al. 2024).

The extraction method in the QuEChERS has been subject to certain variations and modifications to cover a broad range of products, such as the addition of water (for spices, flour, and other dry matrices) or the addition of a buffer method when analyzing pH-sensitive pesticides as suggested by EN 15662 (ECS 2008).

The cleanup approaches are aimed to reducing the matrix effect (ME) in the variability of foods that can be analysed, such as foods with chlorophyll and other natural pigments (e.g., spices), fat or lipid content (e.g., nuts), essential oils and flavonoids (e.g., herbs), etc. (Rutkowska et al. 2019). These MEs lead to analytical problems that affect the accuracy of the results and can be seen in the recovery of pesticides by reducing or increasing the acceptable value for the purpose of validation from 70 to 120% (SANTE 2021; Damale et al. 2023).

One of the most widely used approaches to reduce ME is the matrix-matched calibration method (Cuadros-Rodríguez et al. 2003). Some of its advantages include the ability to utilize a wide range of matrices (Fu et al. 2022; Damale et al. 2023; Rutkowska et al. 2019; Zhao et al. 2021; UNE-CEN/TS-17061, 2019; Kardani et al. 2023). Annex A of the SANTE 11312/2021 (SANTE 2021) document, which describes commodity groups such as high-water content (e.g., vegetables), high acid and high water content (e.g., citrus fruits), high starch and/or protein and low water and fat content (e.g., cereals), or unique commodities such as tea, spices, or coffee, can be used to extrapolate some recommendations for the preparation of matrix calibrations.

For a laboratory analyzing a wide range of products and a large number of samples within the same product group, it is most effective to use a single matrix calibration for different matrices within the same product, which may result in reduced accuracy for some pesticides. In this sense, the design of an analytical Quality Assurance (AQA) system is very important, and the most efficient system for AQA is on-going validation or performance verification with the spike matrix to demonstrate applicability to other commodities in the same commodity group with the same matrix calibration.

Any commercially available chromatography instrument has both acquisition and processing software to analyze AQA requirements, including retention times, sample recovery, blank, calibration, or MS/MS identification. However, these software do not allow an assessment of whether the recovery achieved is within the range of the average recovery and the relative standard deviation (RSD) from on-going recovery results [within laboratory reproducibility (RSD_wR_)]. Another major drawback when working with samples subject to MRLs, which may change over time, is knowing whether the result obtained for a particular pesticide exceeds the MRL, because although this is not defined as a specific AQA criterion, it is a criterion that affects the quality of the product and other criteria such as those defined in section D15 of the guidance “Analytical Quality Control And Method Validation Procedures For Pesticide Residues Analysis In Food And Feed Sante 11312/2021” (SANTE 2021) relating to confirmation of pesticide values exceeding the MRL. In those cases, a confirmatory analysis of another analytical portion is always required.

The great development of information technology in recent years has made it possible to provide commercial AQA software that allows, externally to the instrument software, many statistical and evaluation tools that allow easy and intuitive evaluation of the range of mean recovery and RSD_wR_ through, for example, Shewhart’s charts, which are commonly used for quick graphical visualization of historical data (Agüera et al. 2004; ISO-7870–2, 2023). However, this tool and software also has some disadvantages when used in a multiresidue method to analyze pesticides. For instance, there is a different interpretation of the classic Shewhart’s chart when used in analytical chemistry, as shown in ISO/TS 13530 (ISO/TS-13530, 2009). Two plots are required, one for recovery and the other for precision for each pesticide, and it can be complex to study a multiresidue method with many pesticides (between GC and LC). In addition, other intrinsic characteristics of pesticide analysis, such as the MRLs values or the risk associated with the results of the pesticide versus MRLs, are not considered in this type of chart (IEC 2019; Su et al. 2024; Caldas 2023; Li et al. 2023).

Therefore, the aim of this study is to create and to evaluate an alternative graphical tool to Shewhart´s charts, called Fast Risk Estimation and Analysis (FREA), for multiresidue analysis of pesticides by chromatography and mass detector in a routine laboratory.

The purpose of this graph is to simultaneously display information on the recovery, the RSD_WR_ of the method, and ME for each pesticide and sample analyzed. This will provide insight into the on-going method performance verification and whether the product analyzed exceeds the MRL in the samples through the Index of Quality for Residues (IqR) parameter. This type of graph would enable a quick and visual assessment of the pesticides that have been analyzed with values above the limit of quantification (LOQ), as well as their associated risk. This would be reported in terms of AQA and also by their value in relation to the MRL.

## Materials and Methods

### Sample Selection

The commodity group used was High Water Content (G.HW) because of the wide variability of the different products it contains and the diversity of pesticides and MRLs. The products analyzed were onions and tomatoes, which are shown in Table 1.

**Table 1:** Samples analyzed by commodity group

Blank-spike samples G.HW	Blind samples G.HW	Commodity EURL database (Blind samples)	Product EURL database (Blind samples)
Onions	Onions	0220000: Bulb vegetables	0220020: Onions
Tomatoes	Tomatoes	0230000: Fruiting vegetables	0231010: Tomatoes

G.HW: High water content group; Commodity: Commodity group and code assigned in EURL database; Product: code assigned to the product inside the commodity EURL database

A single matrix-matched calibration using pepper was performed to analyze all these products. Pepper was used because it was previously validated according to the SANTE 11312/2021 (SANTE 2021). The pepper matrix was chosen based on internal validation studies previously carried out, and because it was considered very different from the other matrices to be analyzed (tomato and onion), so that the possible ME could be evaluated (SANTE 2021).

Table 1 also displays the samples categorized as blank-spike and blind, along with their classification from the EURL MRL database. The blank-spike samples were supplied by an accredited laboratory for pesticide analysis in food (ISO 2017) located in the Region of Murcia (Spain), which are usually used by the laboratory as matrix calibration in its routine analyses.

The blind samples were chosen based in the different classifications in the EURL MRL database (EU 2023b) within the commodity group G.HW. These samples were obtained from local companies prior to sale in supermarkets. The sub-sampling and preparation of each G.HW sample were carried out according to R.D. 290/2003 (RD 2003). The homogeneous products obtained were kept in the freezer at − 20 °C until extraction and analysis.

All the samples described in Table 1 were analyzed only once, following the standard procedures of any routine laboratory.

### Chemical and Reagents

All pesticides had certified reference standards of at least 95% purity and were purchased from LGC Standards (Teddington, Middlesex, UK).

Gas chromatography and liquid chromatography solvents [(ethyl acetate, methanol, deionized water (resistivity 18 MΩ), and residual formic acid] were supplied by J.T. Baker (Center Valley, PA, USA). The ammonium formate, formic acid, and acetonitrile were supplied by Merck (Darmstadt, Germany). Sodium chloride (1 g), disodium hydrogen citrate sesquihydrate (0.5 g), trisodium citrate dihydrate (1 g), sodium sulfate (4 g), and primary secondary amine (PSA) for dispersive solid-phase extraction (dSPE) were supplied in an extraction kit by Agilent Technologies (Santa Clara, CA, USA). All reactive were supplied with analytical quality.

### Preparation of Solvent, Matrix Calibration, and Spiked Samples

The stock standard solution was prepared for individual pesticides at a concentration of about 1000 µg/ml. The solvent used for LC was acetone and for GC n-hexane/acetone (9:1, v/v).

The standard working matrix calibration of multiple compounds was performed by serially dissolving the appropriate amounts of each stock solution. The matrix calibration used for G.HW was a blank pepper. The concentrations of the matrix calibration were in the range of 2–50 ng/ml. The fortified samples for the ME study were a white matrix of onion and another of tomato.

### Pesticide Analysis

The QuEChERS extraction, based on EN 15662 (ISO 2019), used 10 g into a 50 ml polypropylene tube without water addition. After, 10 ml of acetonitrile was added [E1 EN 15662 extraction (ISO 2019)], and the mixture was shaken for 1 min. After shaking, the salts extraction kit was added, and it was centrifuged for 5 min at 3000 rpm. A clean-up process using PSA [based in C2 EN 15662 clean-up (ISO 2019)] on the organic aliquot obtained, and finally, it was centrifuged again at 3000 rpm for 5 min to get the final aliquot for GC–MS/MS and LC–MS/MS analysis.

A total of 23 pesticides were analyzed by GC–MS/MS using an Agilent (Santa Clara, CA, USA) 7890A, a Gas Chromatography system coupled with a 7000A quadrupole tandem mass spectrometer. Chromatographic separation was performed on a RTx-5MS column (30 m, 0.25 mm, i.d., 0.5 µm) RESTEK (Agilent, Santa Clara, CA, USA). Helium was used as the carrier gas at a constant flow rate of 1.2 mL/min, while argon was used as the collision gas. The oven temperature was adjusted as follows: The initial temperature was set at 70 °C for 2 min, then increased to 150 °C at a rate of 25 °C min^−1^, to 200 °C at 3 °C min^−1^ with a hold time of 1 min, and finally to 280 °C at 10 °C min^−1^ with a hold time of 10 min. The total run time was 45 min. The temperatures of the transfer line and ion source were set at 280 and 250 °C, respectively. The mass spectrometer was operated in multiple reaction monitoring (MRM) mode with three mass transitions.

A total of 18 pesticides were analyzed by LC–MS/MS using an Agilent liquid chromatography system (Santa Clara, CA, USA) coupled with a 6470 triple quadrupole tandem mass spectrometer. The chromatographic separation was performed on a Poroshell C18 column (150 mm, 2.1 mm i.d., 2.7 μm) (Agilent, Santa Clara, CA, USA) with a flow rate of 0.1 ml at 40 °C. The elution solvent used was water 5 mM ammonium formate with 0.01% formic acid (A) and methanol 5 mM ammonium formate with 0.01% formic acid (B). The gradient elution was carried out as follows: 40% solvent B for 0–5 min, changing to 60% solvent B for 6–12 min, and finishing with 100% solvent B for 17–20 min. Pesticides were analyzed using programmed MRM in positive and negative modes simultaneously. Ion source parameters include optimized drying gas temperature, drying gas flow, nebulizer pressure, sheath gas temperature and flow, capillary voltage, nozzle voltage and high and low radio frequency voltage.

Limit of quantification (LOQ), validation information, MRM transitions, and the retention times for tested pesticides are found in Supplementary Table S1.

The number of pesticides studied using each technique, and the technique used, were chosen based on the history of positive results for the matrices analyzed in this study.

### Performance of the Method and Quality Assurance

The method for pepper was previously validated according to the SANTE 11312/2021 (SANTE 2021) requirements. The blind-incurred samples of G.HW were analyzed in the same sequence or batch according to the quality assurance section titled “on-going method performance verification during the routine analysis” from SANTE 11312/2021 (SANTE 2021) guide. Therefore, together with the sequence of blind-incurred samples, the blank samples spiked at a LOQ of 0.005 mg/kg validated with all pesticides were analyzed.

### Database Creation and Data Analysis

The Structured Query Language (SQL) database is one of the most widely used because it is a free tool and easy to implement in different programming languages (Xia et al. 2003; Strassemeyer et al. 2017). A database of MRLs in the EU was created by downloading information in Extensible Markup Language (XML) from the EU website (EU 2023a).

In the same database, another table was created with information of the data with recoveries for each pesticide in the commodity group studied for automatic RSD_wR_ calculations. Both tables are relational tables linked by the pesticide identifier (ID) present in the XML file from EU web page, and they are periodically updated automatically, allowing both the MRL and the definition of the pesticide to be updated.

The results of the samples analyzed (blind and spike) were exported through the Agilent MassHunter software (Anonymous 2008) in an XML format to a processing computer code in Python (3.11.4) or PHP 7.0 language using open libraries for data analysis.

Both the Python and PHP code generate hypertext markup language (HTML) with graphics through the open-source Google Chart (Google 2023b). The graphical option selected to generate a FREA chart was the bubble chart (Google 2023a).

The figures generated in HTML code that appear in this manuscript, as well as the source code used in PHP, together with the SQL databases, can be obtained on request from the authors and the author´s GitHub repository.

### AQA Data Analysis

The quality control based on the check of the routine recovery (Rec) of the spiked samples was calculated using Eq. 1.

1 $$\% {\text{Rec = }}\frac{{{\text{Measured}}\;{\text{concentration}}}}{{{\text{Spiked}}\;{\text{concentration}}}} \times {100}$$

where measured concentration is the concentration for each blind-incurred sample and each pesticide. Spiked concentration is the theoretical concentration spiked, 0.005 mg/Kg in this case.

The quality results, based on the reproducibility on-going method, are given by the Relative Standard Deviation (RSD_wR_) of all the running verification samples (8 historical data) together the spiked samples analyzed in the same time and batch, by Eq. 2.

2 $$\% {\text{RSD = }}\frac{{{\text{Standard }}\;{\text{deviation}}}}{{\text{Average }}} \times 100$$

where the standard deviation and mean are the results of calculating the standard deviation and mean for each historical routine recovery data for each pesticide together with the current recovery in the batch analyzed.

The limits for both calculations are given in section C43 of the SANTE Guide (SANTE 2021). For routine analysis, a practical default of 60–140% can be used for individual recoveries, but with a maximum RSD of 20%.

To assess the quality of the food in the samples analyzed, the index of quality for residues (IqR) could be used (Mac Loughlin et al. 2018; Bibi et al. 2022) as shown in Eq. 3.

3 $$IqR=\frac{PRC}{MRL}$$

where PRC is the pesticide residue concentration (mg/kg) in the blind sample. The results thus obtained for IqR could be evaluated as good (0–0.6), adequate (0.6–1.0), and inadequate (> 1).

**Fig. 1:**
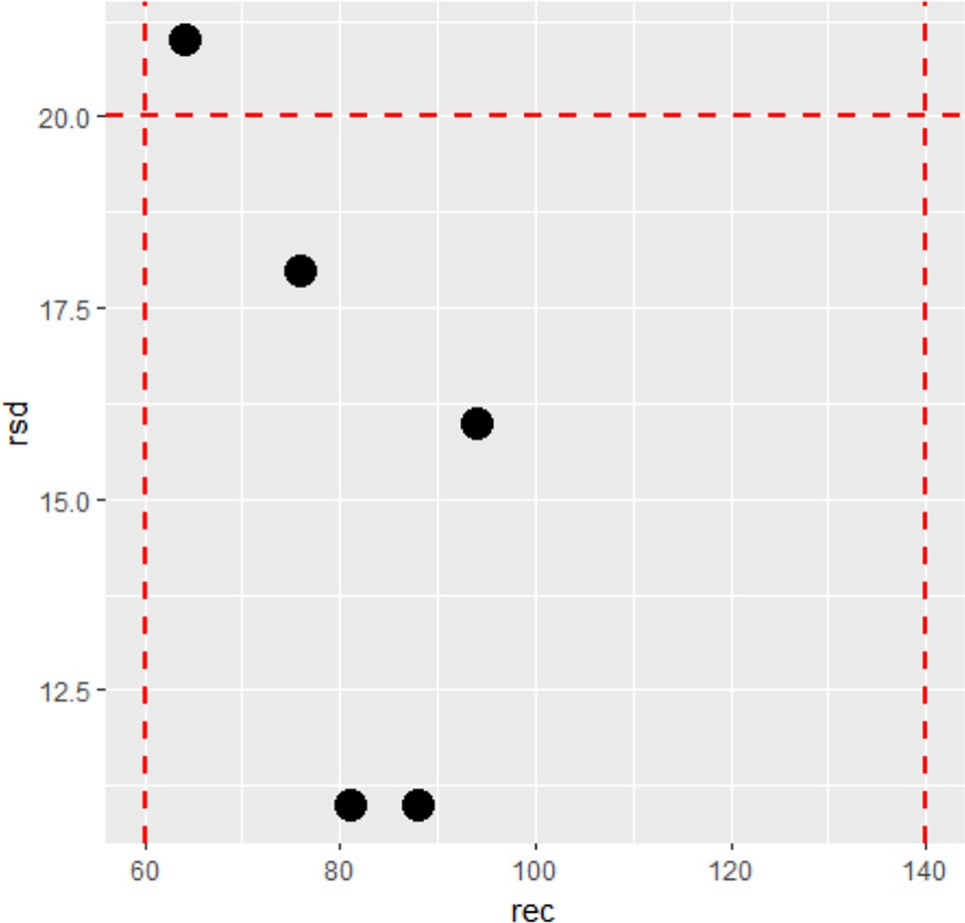
Chart considering RSD_wR_ a recovery in routine analysis

The combination of the three equations gives information about the analytical compliance. These three variables were combined in a bubble chart. Figure 1 shows a bubble chart with data of different pesticides (each black bubble), recovery values (axis x), and RSD_wR_ values (axis y). The diameter of the bubble indicates the value of IqR.

When validating a method for analyzing multiple pesticide residues, it is important to ensure that the limit of quantification is suitable for the maximum residue level (MRL) of each pesticide. Additionally, some pesticides have complex definitions that require validation of different compounds. For example, carbofuran—though not analyzed in this study—has an MRL set for various compounds and metabolites. This includes any carbofuran formed from substances such as carbosulfan, benfuracarb, or furathiocarb, as well as 3-OH carbofuran, all expressed as total carbofuran.

However, these variables may change rapidly depending on the changes introduced by Regulation 396/2005 (EC 2005), such as the introduced value of IqR (IqRm) to add additional evaluations about the analytical compliance in the pesticide analysis. Equation 4 shows the modifications to identify two different cases:

4 $$IqR_{m} = \frac{{PRC_{m} }}{MRL } \times 100\;\;\;{\text{IqR}}_{{\text{m}}} = \left\{ {\begin{array}{*{20}l} {{\text{Case}}\;{1:}\;{\text{ MRL < Spiked}}\;{\text{concentration}}} \hfill \\ {{\text{Case}}\;{2:}\;{\text{MRL}}\;{\text{complex}}\;{\text{definitions}}} \hfill \\ \end{array} } \right.$$

Case 1:

Specific pesticides in diverse products present MRLs lower than the LOQ, e.g. carbofuran in wine grapes has a MRL of 0.002 mg/kg. Also, the MRLs values may be modified in the future changes to the Regulation 396/2005 (EC 2005). Updating XML files in the database allows detect MRLs < spiked concentration and shows a 150% result of IqR_m_.

Case 2:

Some of pesticide are expressed and quantified through a single component (e.g.' oxyfluorfen), but other pesticides have a complex residue definition because the MRL is based in the sum of more of one component that is analyzed separately (e.g., endosulfan MRL is based in the analysis of alpha endosulfan, beta-endosulfan, and endosulfan sulfate). In these cases, only to evaluate the risk with the MRL value, it is necessary to consider that each component must comply with the MRL or use the residue definition in a specific manner taking into account the molecular weight conversion factor (EC 2015). In the case of a complex definition, IqRm uses the MRL of the sum for each component.

All these cases were represented on different colored bubbles, as shown in the following scheme:

$${\text{Bubble chart}} = \left\{ {\begin{array}{*{20}l} {{\text{Green: }}\;{\text{IqRm}}\,{ < }\,{60}\% \;{\text{and}}\;{\text{RSD}}\,{ < }\,{20}\,\% \;{\text{and}}\;{\text{recovery }}\;{\text{between}}\;{60}\% { - 140}\% } \hfill \\ {{\text{Yellow:}}\;{\text{IqRm}}^{3} {60}\% \;{\text{and}}\;{ < }\,{100}\% \;{\text{and}}\;{\text{RSD}}\;{ < }\,{20}\% \;{\text{and}}\;{\text{recovery}}\;{\text{between}}\;{60}\% { - 140}\% } \hfill \\ {{\text{Red:}}\;{\text{IqRm}}\,{ > }\;{100}\% \;{\text{or}}\;{\text{RSD > 20}}\% \;{\text{or}}\;{\text{recovery}}\,\left\langle {{60}\% \,{\text{or}}} \right\rangle \,{140}\% } \hfill \\ {{\text{Grey:}}\,{\text{LMR}}\,{ < }\,{\text{spike}}\;{\text{concentration}}\;{\text{or}}\;{\text{(ME > 20}}\% \;{\text{and}}\;{\text{at}}\;{\text{least}}\;{\text{two}}\;{\text{other}}\;{\text{non - compliance)}}} \hfill \\ \end{array} } \right.$$

The scheme shows the different criteria used to plot the bubble chart. Note that for gray bubble chart could be for a non-compliance for at least two non-compliance about the IqRm, RSD_wR_, or recovery.

The ME could be estimated by comparing the slopes of matrix-matched calibration curves with the solvent calibration curves (Damale et al. 2023; de Sousa et al. 2012). However, this presents a big problem for the analytical effort (cost and time) involved for each product in the same commodity group. For these reasons, an alternative calculation (Eq. 5) to evaluate ME was proposed.

5 $${\text{ME = }}\frac{{{\text{Absolute}}\;{\text{(Rec}}_{{1}} {\text{ - Rec}}_{{2}} {)}}}{{{\text{(Rec}}_{{1}} {\text{ + Rec}}_{{2}} {)/2}}} \times {100}$$

The ME proposed calculates the relative percentage difference (RPD) (Anonymous 2017) between two different recoveries of the two different spike samples in the routine batch analyzed. In this study, Rec1 is the recovery of the tomato spike sample, and Rec 2 is the recovery obtained from the onion spike. A maximum value for RPD of 20% was fixed, by similarity, to the value of RSD_wR_ fixed in SANTE 11312/2021 (SANTE 2021).

## Results and Discussion

Table 2 shows the results of pesticide analysis in blind samples of tomatoes and onions, conducted through GC and LC analysis. The concentrations are expressed with two significant figures according to SANTE 11312/2021 (SANTE 2021) for all results over of the LOQ of 0.005 mg/kg. Table 2 also shows the MRL for both samples.

**Table 2:** Pesticide results and MRL values

Pesticide	Analysis	mg/kg (Onion)	mg/kg (Tomato)	MRL (Onion)	MRL (Tomato)
Chlorpropham	GC	0.053	< 0.005	0.01	–
Fenpropathrin	GC	0.095	< 0.005	0.01	–
Fluopyram	LC	0.079	0.090	0.07	0.5
Mandipropamid	LC	0.0083	< 0.005	0.1	–
Spirotetramat (sum)	LC	0.078	0.072	0.4	1
Spirotetramat enol	LC	0.097	0.089	–	–
Pendimethalin	GC	0.071	< 0.005	0.05	–
Acetamiprid	LC	< 0.005	0.028	–	0.5
Azoxystrobin	LC	< 0.005	0.0082	–	3
Boscalid	LC	< 0.005	0.043	–	3
Chlorantraniliprole	LC	< 0.005	0.012	–	0.6
Cypermethrin	GC	< 0.005	0.185	–	0.5
Cyproconazole	GC	< 0.005	0.024	–	0.05
Cyprodinil	GC	< 0.005	0.107	–	1.5
Dimethomorph	GC	< 0.005	0.031	–	1
Fenhexamid	LC	< 0.005	0.049	–	2
Fludioxonil	GC	< 0.005	0.025	–	3
Iprodione	GC	< 0.005	0.036	–	0.01
Metaflumizone	LC	< 0.005	0.039	–	0.7
Pyraclostrobin	LC	< 0.005	0.013	–	0.3
Pyriproxyfen	GC	< 0.005	0.279	–	1
Spinosad	LC	< 0.005	0.045	–	0.7
Spirodiclofen	LC	< 0.005	0.026	–	0.5
Spiromesifen	LC	< 0.005	0.055	–	1
Thiacloprid	LC	< 0.005	0.049	–	0.5

Spirotetramat (sum): Spirotetramat and spirotetramat-enol (sum of), expressed as spirotetramat: Spinosad: Spinosad (spinosad, sum of spinosyn A and spinosyn D); Cypermethrin: Cypermethrin [cypermethrin including other mixtures of constituent isomers (sum of isomers)]; Conc: Concentration in mg/kg; MRL: Maximum Residue Level from EURL database; –: MRL value not shown due to the current lack of MRL values in the EURL database: therefore, the default value of 0.01 mg/kg can still be used

The number of pesticides with values above the LOQ or reported as positive was 7 in the onion sample and 21 in the tomato sample. The number of positive pesticides in tomato and onions was similar in number to other pesticide studies in these matrices (Jirata et al. 2024; Ouakhssase and Ait Addi 2023). The number of pesticides with values in above the LOQ in onions is much lower than in tomatoes, which could be due to the inhibitory effect of onion organosulfur compounds (propylpropane thiosulfinate and propylpropane thiosulfonate) on many pests (Falcón-Piñeiro et al. 2023; Skovgaard et al. 2017) and mainly because they are bulbs (grown under the soil).

The three highest values for onion correspond to the pesticides fluopyram (0.079 mg/kg), fenpropathrin (0.095 mg/kg), and spirotetramat (0.097 mg/kg). In the case of spirotetramat, the value is obtained exclusively from the result of spirotetramat enol. As can be observed, all pesticides are below the MRL due to spirotetramat, even though it is a pesticide belonging to the derivatives of tetronic acid, it has a low persistence (Mandal et al. 2022). However, in the case of fluopyram, it is above the MRL due to a greater persistence of the compound (Patel et al. 2016).

In the case of tomato, the three pesticides found were cyprodinil (0.279 mg/kg), cypermethrin (0.185 mg/kg) and pyriproxyfen (0.107 mg/kg). These pesticides are commonly used in tomatoes, but they have low persistence due to factors such as photodegradation (Lin et al. 2005). In the case of cypermethrin, the results are the sum of different chromatographic peaks automatically via software in GC analysis (Khazri et al. 2016).

Table 3 shows the data calculated of the spiked samples of onion and tomato (blank samples) such as % of recovery (%Rec), % RPD (as estimation of ME) described by Eq. 5, historical data for %RSD in the commodity group studied (G.HW), and the results for the IqRm for all the pesticides analyzed by GC and LC. Of note, pesticide concentrations in the blank matrix were not included in Table 3 because they were below the LOQ (< 0.005 mg/kg) in all cases.

**Table 3:** Calculations obtained for the generation of FREA chart

Pesticide	%Rec (Onion)	%Rec (Tomato)	%RPD (ME)	%RSD (G.HW)	IqR_m_ (Onion)	IqR_m_ (Tomato)
Chlorpropham	64	86	29	23	530	0
Fenpropathrin	106	70	41	17	950	0
Fluopyram	89	104	16	22	113	0
Mandipropamid	109	75	37	19	8	0
Spirotetramat (sum)	115	91	23	20	20	0
Spirotetramat enol	115	91	23	20	24	0
Pendimethalin	116	103	12	18	142	0
Acetamiprid	106	87	20	17	0	6
Azoxystrobine	102	85	18	13	0	0
Boscalid	77	85	10	15	0	1
Chlorantraniliprole	111	80	32	22	0	2
Cypermethrin	87	108	22	17	0	37
Cyproconazole	113	90	23	13	0	48
Cyprodinil	105	89	16	9	0	7
Dimethomorph	92	69	29	16	0	3
Fenhexamid	81	107	28	18	0	2
Fludioxonil	96	109	13	21	0	1
Iprodione	99	115	15	10	0	360
Metaflumizone	99	83	18	15	0	6
Pyraclostrobin	78	91	15	16	0	4
Pyriproxyfen	94	84	11	14	0	28
Spinosad	118	115	3	15	0	6
Spirodiclofen	88	67	27	19	0	5
Spiromesifen	71	119	51	13	0	6
Thiacloprid	86	62	32	24	0	10

Spirotetramat (sum): Spirotetramat and spirotetramat-enol (sum of), expressed as spirotetramat: Spinosad: Spinosad (spinosad, sum of spinosyn A and spinosyn D); Cypermethrin: Cypermethrin [cypermethrin including other mixtures of constituent isomers (sum of isomers))]

As shown in Table 3, there are four pesticides in the onion with not adequate IqR_m_, but only the chlorpropham and fluopyram present a non-compliance in the RSD, according to the requirements fixed in Eq. 5. However, in the tomato sample, only iprodione presented a not adequate IqRm but all other requirements are fulfilled.

The ME effect, calculated with the RPD using Eq. 5, shows that there are 12 (48%) pesticides with a non-compliance value upper of the LOQ. This could indicate that there is a ME to be considered when using pepper as matrix calibration in tomato or onion since the effects of signal suppression and co-extracted compounds can have a significant effect on the results (Gómez-Ramos et al. 2016; Wu and Ding 2023). However, even taking into account the possible matrix effects, only the pesticides chlorpropham and fenpropathrin represent a high risk in onions due to the concentration of the pesticide with respect to the MRL that using Eq. 4 gives a very high value of the IqRm.

**Fig. 2:**
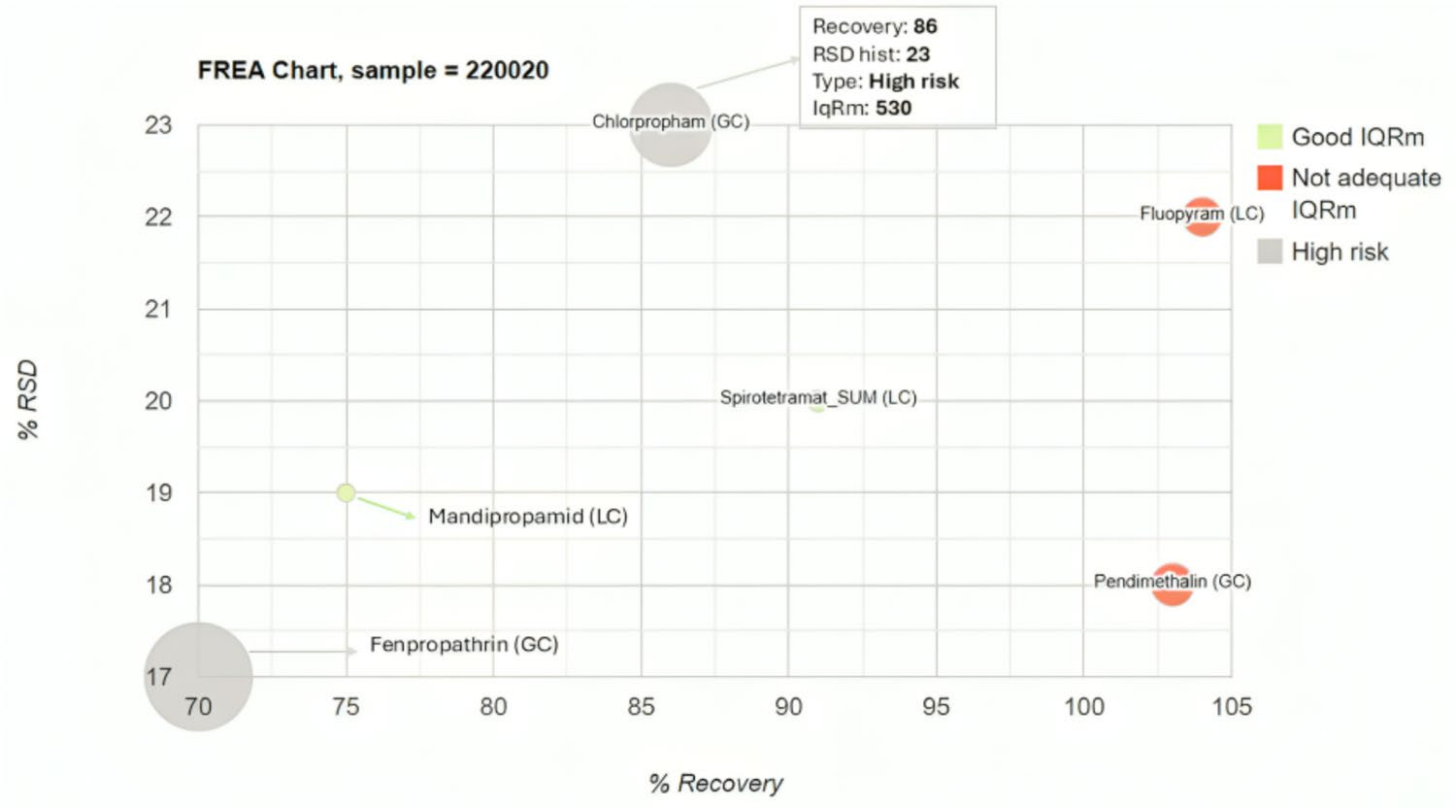
FREA chart for onion sample (only pesticides with values > LOQ from Table 2)

As mentioned earlier, the FREA chart can be generated in two different ways depending on the objective pursued. Figure 2 illustrates how a graph can display information related to pesticides with values above the limit of quantification in a specific sample. These values must be evaluated from the perspective of both the AQA data analysis and the associated risk of that value against the MRL of the pesticide in that sample.

In Fig. 2, it is evident that the gray bubbles, representing chlorpropham, pose a high risk due to non-compliance in RSD, ME, and a larger radius indicating a high IqRm value. On the other hand, the position of fluopyram, the red bubble in the top right-hand corner of Fig. 2, indicates a historical RSD > 20%, but it does not exhibit EM and has a > 100 value of the IqRm. However, its lower radius suggests that the quantified value of the pesticide compared to the MRL is lower in this pesticide than in chlorpropham.

The FREA chart also allows numerical information to be displayed by hovering over each of the bubbles, as shown for the pesticide chlorpropham.

**Fig. 3:**
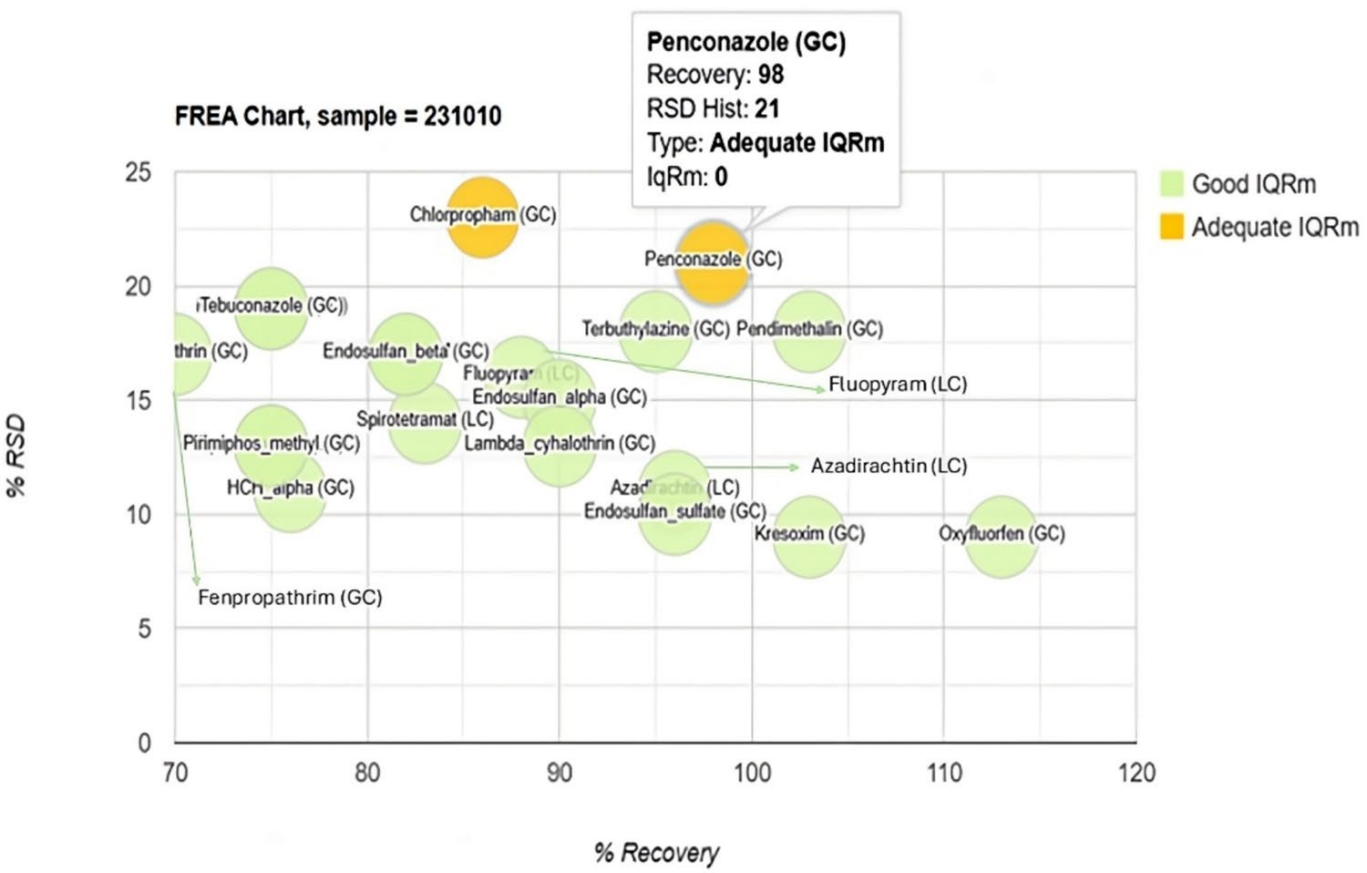
FREA chart for tomato sample (pesticides with values < LOQ from Table 2)

An alternative to displaying positive pesticides as shown in Fig. 2 is to display pesticides with values below the LOQ as shown in Fig. 3. In this way, the AQA criteria can be evaluated only and simultaneously. In Fig. 3, those pesticides with adequate recovery values (60–140%) and RSD (< 20%) appear in green and in yellow when there is a non-compliance, as in the case of penconazole that does not comply with the RSD value by having a value of 21%.

## Conclusions

The results of this study indicate that the FREA chart enables rapid visualization of a larger number of pesticides along with the classical on-going parameters RSDwR and recovery simultaneously.

Using the FREA chart, it was easy to see that only four pesticides in the onion exceeded the limit of quantification analyzed by GC–MS/MS and LC–MS/MS. These pesticides are chlorpropham (IqRm of 530), fenpropathrin (IqRm of 950), fluopyram (IqRm of 113), and pendimetalin (IqRm of 142). This poses a risk that needs to be reviewed as the values in the sample exceeded the MRL. Hovering over the graph, it was observed that chlorpropham had an RSD value of 23% and an ME of 29%, which violates the AQA requirements. For fenpropathrin, only the ME (41%) was found to be non-compliant. In the case of fluopyram, non-compliance was shown for an RSD value over 20% (22%), while pendimetalin did not have any non-compliance issues.

The FREA chart also showed its usefulness by indicating those pesticides that, although they were not positive in the samples, presented some type of non-compliance in the AQA as was the case with chlorpropham and penconazole with RSD values > 20%.

Using the information from the MRL and the periodic updates of the existing databases, the IqRm index allows graphical visualization both to assess the quality of the sample analyzed and to detect changes in the MRL or the definition of the pesticide that may affect the LOQ. This graph also allows to estimate the ME by RPD calculation between two recoveries of different samples analyzed in the batch. However, it has the limitation that it is only evaluated in a single concentration and sample, and a validation in a specific product or alternative solutions could be necessary (Kwon et al. 2012).

This type of graph can be very helpful in making decisions about the analysis of pesticides, as well as determining the risk of a pesticide being present at a concentration above the LOQ, based on the concentration obtained and the MRL in place at the time for the specific type of sample being analyzed.

## Supplementary Information

Below is the link to the electronic supplementary material.

Supplementary file1 (DOCX 30 KB)
