# Supplementary material for: Quick Monitoring of Tomato and Onion Samples During Routine Regulatory Analysis of Pesticide Residues
Source: Arch Environ Contam Toxicol. 2025 Jun 14;89(1):23–33. doi: 10.1007/s00244-025-01133-w (PMC12370558; doi:10.1007/s00244-025-01133-w)
Supplement: Supplementary file 1 — Supplementary file1 (DOCX 30 KB) [file 244_2025_1133_MOESM1_ESM.docx]

**Quick monitoring of tomato and onion samples during routine regulatory analysis of pesticide residues**

José Manuel Veiga-del-Baño ^1^, José Oliva ^1^, Miguel Ángel Cámara ^1^, Pedro Andreo-Martínez ^1,^*, Miguel Motas ^2^

^1^ Department of Agricultural Chemistry, Faculty of Chemistry, Regional Campus of International Excellence “Campus Mare Nostrum”, University of Murcia, Campus of Espinardo, 30100 Murcia, Spain.

^2^ Department of Toxicology, Faculty of Veterinary, Regional Campus of International Excellence “Campus Mare Nostrum”, University of Murcia, Campus of Espinardo, 30100 Murcia, Spain

*Corresponding author: Pedro Andreo-Martínez. E-mail: pam11@um.es

Table S1. Pesticide validation summary detected in samples analyzed.

| Pesticide | Numb | Analysis | MRM | Rec  % | RSD  % | LoQ (mg/Kg) |
| --- | --- | --- | --- | --- | --- | --- |
| Chlorpropham | 5 | GC | 213>171  213>127 | 85 | 12 | 0.005 |
| Fenpropathrin | 5 | GC | 265<210  265>89 | 87 | 11 | 0.005 |
| Fluopyram | 5 | LC | 397>208  397>173 | 85 | 9 | 0.005 |
| Mandipropamid | 5 | LC | 412>328  412>125 | 90 | 13 | 0.005 |
| Spirotetramat | 5 | LC | 374>330  374>302  302>216  302>115 | 81 | 8 | 0.005 |
| Pendimethalin | 5 | GC | 252>162  252>191 | 88 | 14 | 0.005 |
| Acetamiprid | 5 | LC | 223>126  223>90 | 104 | 11 | 0.005 |
| Azoxystrobine | 5 | LC | 404>372  404>344 | 94 | 13 | 0.005 |
| Boscalid | 5 | LC | 343>307  343>271 | 95 | 7 | 0.005 |
| Chlorantraniliprole | 5 | LC | 484>194  484>185 | 85 | 13 | 0.005 |
| Cypermethrin | 5 | GC | 181>152  181>127 | 88 | 12 | 0.005 |
| Cyproconazole | 5 | GC | 222>125  222>82 | 97 | 7 | 0.005 |
| Cyprodinil | 5 | GC | 224>208  224>118 | 105 | 13 | 0.005 |
| Dimethomorph | 5 | GC | 301>165  301>139 | 103 | 13 | 0.005 |
| Fenhexamid | 5 | LC | 302>97  302>55 | 89 | 12 | 0.005 |
| Fludioxonil | 5 | GC | 248>154  248>127 | 83 | 12 | 0.005 |
| Iprodione | 5 | GC | 314>245  314>56 | 82 | 12 | 0.005 |
| Metaflumizone | 5 | LC | 505>285  505>328 | 93 | 7 | 0.005 |
| Pyraclostrobin | 5 | LC | 388>194  388>161 | 80 | 12 | 0.005 |
| Pyriproxyfen | 5 | GC | 136>96  136>78 | 98 | 14 | 0.005 |
| Spinosad | 5 | LC | 732>142  732>98  742>142  742>98 | 85 | 9 | 0.005 |
| Spirodiclofen | 5 | LC | 313>213  313>157 | 100 | 14 | 0.005 |
| Spiromesife | 5 | LC | 273>255  273>187 | 92 | 12 | 0.005 |
| Thiacloprid | 5 | LC | 253>126  253>90 | 87 | 9 | 0.005 |

Analysis: GC corresponding to GC-MS/MS analysis, LC corresponding to LC-MS/MS analysis; Numb: Number of repetitions for the pesticide validation in a representative matrix (pepper) for the commodity group G.HW; MRM: precursor ion > product ion (quantitative), precursor ion > product ion (qualitative). In the case of spirotetramat and spinosad, the table show the ions for spirotetramat and spirotetramat enol and spinosyn A and spinosyn D; %Rec: Average recovery percentage for each pesticide in the validation process.; %RSD: Relative standard deviation for each pesticide; LoQ: Limit of Quantification calculated with 5 blank spike samples of pepper with a recovery between 70-120% and RSD<20% according to SANTE 11312/2021.
